# What differences are detected by superiority trials or ruled out by noninferiority trials? A cross-sectional study on a random sample of two-hundred two-arms parallel group randomized clinical trials

**DOI:** 10.1186/1471-2288-10-93

**Published:** 2010-10-15

**Authors:** Angèle Gayet-Ageron, Thomas Agoritsas, Christophe Combescure, Krisztina Bagamery, Delphine S Courvoisier, Thomas V Perneger

**Affiliations:** 1 Division of Clinical Epidemiology, University Hospitals of Geneva and Faculty of Medicine, University of Geneva, Geneva, Switzerland

## Abstract

**Background:**

The smallest difference to be detected in superiority trials or the largest difference to be ruled out in noninferiority trials is a key determinant of sample size, but little guidance exists to help researchers in their choice. The objectives were to examine the distribution of differences that researchers aim to detect in clinical trials and to verify that those differences are smaller in noninferiority compared to superiority trials.

**Methods:**

Cross-sectional study based on a random sample of two hundred two-arm, parallel group superiority (100) and noninferiority (100) randomized clinical trials published between 2004 and 2009 in 27 leading medical journals. The main outcome measure was the smallest difference in favor of the new treatment to be detected (superiority trials) or largest unfavorable difference to be ruled out (noninferiority trials) used for sample size computation, expressed as standardized difference in proportions, or standardized difference in means. Student t test and analysis of variance were used.

**Results:**

The differences to be detected or ruled out varied considerably from one study to the next; e.g., for superiority trials, the standardized difference in means ranged from 0.007 to 0.87, and the standardized difference in proportions from 0.04 to 1.56. On average, superiority trials were designed to detect larger differences than noninferiority trials (standardized difference in proportions: mean 0.37 versus 0.27, *P *= 0.001; standardized difference in means: 0.56 versus 0.40, *P *= 0.006). Standardized differences were lower for mortality than for other outcomes, and lower in cardiovascular trials than in other research areas.

**Conclusions:**

Superiority trials are designed to detect larger differences than noninferiority trials are designed to rule out. The variability between studies is considerable and is partly explained by the type of outcome and the medical context. A more explicit and rational approach to choosing the difference to be detected or to be ruled out in clinical trials may be desirable.

## Background

A key step in planning a randomized clinical trial is the determination of the smallest difference in the primary outcome that should be detected between the study arms. This difference determines the sample size to be used in the study together with the type I error, power and variance of the primary outcome. In principle, this determination should be made a priori by the researchers [1] based on scientific and public health arguments; only then should the sample size be calculated. In reality, researchers often start with a small difference to be detected, which they subsequently revise until an achievable sample size is obtained [2,3]. The danger of such practice is that trials may end up being sufficiently powered to detect convenient differences, but underpowered to detect clinically meaningful differences. On the other hand, there is currently no objective, scientific method for determining the smallest difference that is important for Science and Society. Given this uncertainty, the description of current practice can provide a useful framework for judging what differences are large or small.

Stating the smallest difference to be detected is necessary in planning a superiority trial. Planning an equivalence or noninferiority, trial (henceforth called noninferiority trial) requires a different input: the largest unfavorable difference that is still compatible with noninferiority. Logically, a noninferiority margin should be smaller than a superiority margin, other things being equal, since the former is compatible with equality between treatments, while the latter excludes equality. A noninferiority margin that is too wide may lead to the conclusion that a new treatment is equivalent to the standard treatment when in fact it is inferior. Of note, an alternative approach exists for testing noninferiority in the main outcome at the same time as superiority in a secondary outcome, such as safety or convenience [4].

There is no consensus regarding the procedures for choosing a difference to be detected or ruled out. In contrast, the type I error rate is often set to 5% and study power to 80% or 90%. Thus the superiority or noninferiority margin is the main cause of variability in sample size between trials. In turn, sample size strongly influences the feasibility and cost of a trial. Surprisingly little is currently known about the distribution and determinants of differences to be detected or ruled out in trials.

Our main goal was to verify whether superiority margins used in planning clinical trials are indeed larger on average than non-inferiority margins used in planning noninferiority trials, and to examine the variability between studies in these parameters. Our second goal was to identify study-related factors that may influence the choice of the difference, such as the nature of the study outcome, the clinical field, the type of treatments compared, etc.

In this article, we reported on a survey of the clinical important differences used by researchers to estimate their sample size in 100 randomly selected superiority trials and 100 noninferiority trials published in 27 leading medical journals between January 2004 and March 2009. In the "Methods" section, we describe our search strategy. The data we provide reflects that superiority trials are designed to detect larger clinical differences and noninferiority trials ruled out smaller differences; the variability observed is considerable and may be partly explained by the medical context and the type of outcome.

## Methods

### Study design and sample

We conducted a cross-sectional study based on 200 randomized clinical trials published between January 1^st^, 2004 and March 1^st^, 2009. We aimed to assess a priori clinical difference among high-quality, clinically relevant studies in internal medicine, general practice and mental health [4]. We therefore performed a search in Medline (Pubmed) using the following terms [4,5]: "randomized controlled trial" OR ("randomized" AND "controlled" AND "trial"), within publication types, subject headings or text words and restricted our search to trials published in 27 medical journals with high impact factors (Additional file 1). In a second step, in order to retrieve a sufficient number of noninferiority trials, we added the keywords "non-inferiority" OR "equivalence" to the search. We recorded all retrieved citations in a SPSS database then screened full texts of the articles for eligibility. Among the retrieved studies, we randomly selected 200 trials.

### Eligibility criteria

We included only two-arm, parallel group trials with a single primary outcome that could be either a continuous or a binary variable. We excluded crossover trials and cluster-randomized trials which yield paired data and require specific calculations to estimate the sample size. We also excluded nonrandomized trials mislabeled as randomized trials, ancillary analyses of previously published studies, and studies that used time-to-event variables as outcomes. Two readers (AGA, KB) verified inclusion and exclusion criteria, and for eligible papers, extracted relevant data. Uncertainties were discussed and discrepancies in the assessment of relevant articles were resolved by consensus.

### Data extraction

From the full published report, we recorded the journal, the year of publication, the medical specialty, the interventions compared (pharmacological, vaccine, surgical, medical devices and strategies, including diagnostic, medical care management, rehabilitative interventions), the type of primary outcome (dichotomous, continuous) and whether it was related to mortality or not, whether the trial was multicenter or not, whether a research methodologist (statistician or epidemiologist) was associated (retrieved from the authors' affiliation list and acknowledgement section), whether the trial was supported by industry, or by another source of funding (institutional or private grant) or lacking any financial support. We also classified trials in four subgroups based on the targeted study population: children below 18 years, mother and child, adults, or elderly. Finally we made subgroups on the major medical context (cardiovascular, infectious diseases or oncology versus other medical specialties).

Since all articles were published after the revised CONSORT statement in 2001 [6-8], details of a priori sample size computation should always be reported. We collected parameters used for this calculation: type I error, one or two-tailed test, type II error, and estimated sample size. For dichotomous outcomes, we retrieved event proportions in the control and active group (P_1_, P_2_) or treatment effect of interest (difference of proportions). For continuous outcomes, we retrieved the difference in means (m_2 _- m_1_), and standard deviation, or the effect size.

### Outcomes

When outcomes were expressed as proportions, we calculated a standardized difference in proportions as (P_2 _- P_1_)/√(P(1-P)) where P is the weighted mean of P_1 _and P_2_. This index is analogous to that used for contrasting two means [9]. When the difference in proportions was available but proportions in the treatment arms (P_1_, P_2_) were lacking, P_1 _and P_2 _were recalculated using the formulae for sample-size calculation adapted for a χ^2 ^test or Fisher's exact test or those adapted for bioequivalence trials [10,11].

For continuous variables, we calculated the standardized difference in means as the difference of means divided by the pooled standard deviation: (m_2 _- m_1_)/SD. When the standard deviation was not given in the methods section, we used the standard deviation described in the results section and verified the sample size using formulae for the Student's *t *test or for bioequivalence trials [10,11]. In analyses that pooled the two types of studies, we used the standardized difference in outcomes, regardless of the type of outcome.

### Independent variables

The main predictor was the type of the trial: superiority versus noninferiority. Other independent variables were: mortality (single or composite outcome) or not, medical context (cardiovascular, infectious diseases, oncology, or other medical specialties), age-group of the study population (neonates and children below 18 years of age, adults and elderly), type of intervention (pharmacological versus other), involvement of a statistician/epidemiologist, year of publication (2004-06 versus 2007-09), funding source (industry, institution versus no fund or not stipulated) and finally a single-center or multicenter recruitment of participants. The target sample size (≤200, 200-400, 400-800 or >800 participants defined following quartiles) was also studied in relation to standardized differences, even though sample size is a consequence of this difference, not its determinant.

### Sample size estimation

We sought to detect a moderate difference of the mean standardized differences between superiority and noninferiority trials, which we defined as half a standard deviation [12]. This difference would require 84 trials per group using a power of 90% and a type I error of 5% (two-tailed). We rounded-off the sample size to 100 superiority and 100 noninferiority trials.

### Statistical analysis

We first compared the characteristics of the included superiority and noninferiority trials, using the Mann-Whitney test for continuous variables and Chi-square or Fisher's exact test for categorical variables. Then we examined the distributions of the standardized differences in superiority and noninferiority trials, and compared their means using the Student *t *test. We performed subgroup analyses, separately for superiority and noninferiority trials, comparing the standardized differences according to various study characteristics using *t *tests or one-way analysis of variance. We also tested for interaction between the main effect (superiority/noninferiority type of the trial) and each factor; if the interaction was not significant, a single *P*-value for the factor was presented in the last column of the corresponding Table. Finally we used an analysis of variance assessing the variance of the standardized differences by several predictors of interest identified in this preliminary analysis of variance (noninferiority versus superiority trials, mortality versus nonmortality trials and the medical context) and we presented the estimated mean standardized difference, its standard error and the associated *P*-value.

All analyses were performed using SPSS version 17.0 (SPSS Inc., Chicago, Illinois, USA) and Stata IC 11 (STATA Corp., College Station, Texas, USA). Statistical significance was defined as *P *< 0.05 (two sided).

## Results

### Selection of articles

**Figure 1:**
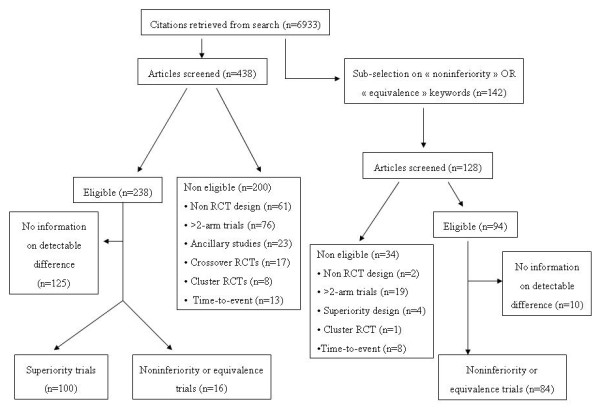
Flow chart of published randomized-controlled trials considered for inclusion.

The initial search yielded 6933 citations. We randomly selected a total of 580 articles in order to obtain our goal sample of 200 articles that contained information on the difference to be detected or ruled out (Figure 1). Of note, for 135 (40.7%) among 334 eligible trials, there was no value in full reports that described the difference to detect. Additional file 2 lists the 100 superiority and 100 noninferiority trials included.

### Trial characteristics

Superiority and noninferiority trials did not differ in the journal of publication (Table 1). Justification for the choice of the treatment difference used was found at a significantly higher rate in superiority compared to noninferiority trials. Noninferiority trials dealt more often with infectious diseases, examined pharmacologic interventions, and used dichotomous outcomes more often than superiority trials. Superiority trials were mainly conducted in other medical context than cardiovascular, infectious diseases or oncology (neuro-psychiatry, n = 17; rheumatology, n = 6; internal or general medicine, n = 4; medical education, n = 3; other, n = 47). Noninferiority trials required larger sample sizes, were more often conducted at multiple centers than superiority trials and were more often funded by the industry.

**Table 1:** Characteristics of the 200 articles included.

Characteristics	Superiority trials (n = 100)	Noninferiority trials (n = 100)	P-value*
Journals, No. (%)			0.24**
N Engl J Med	14 (14)	21 (21)	
J Clin Oncol	5 (5)	8 (8)	
JAMA	6 (6)	10 (10)	
Pediatrics	11 (11)	7 (7)	
Lancet	10 (10)	4 (4)	
Other	54 (54)	50 (50)	

Years of publication, No. (%)			0.99
2004-2006	59 (59)	59 (59)	
2007-2009	41 (41)	41 (41)	

Justification for the treatement difference, No (%)			0.02
Yes (by published or pilot studies)	59 (59)	49 (49)	
No	41 (41)	61 (61)	

Medical specialty, No (%)			<0.001
Cardio-vascular diseases	14 (14)	19 (19)	
Infectious diseases	3 (3)	23 (23)	
Oncology	6 (6)	10 (10)	
Other	77 (77)	48 (48)	

Intervention, No. (%)			0.02
Pharmacological	54 (54)	73 (73)	
Surgery	8 (8)	3 (3)	
Strategy (diagnosis or management)	34 (34)	18 (18)	
Other	4 (4)	6 (6)	

Patient categories, No. (%)			0.18**.
Adults (including elderly)	77 (77)	82 (82)	
Neonates and children < 18 yrs	18 (18)	18 (18)	
Mother and child	5 (5)	0 (-)	

Primary outcome, No. (%)			<0.001
Dichotomous	60 (60)	86 (86)	
Continuous	40 (40)	14 (14)	

Type of outcome, No. (%)			0.17
Mortality	12 (12)	19 (19)	
Other	88 (88)	81 (81)	

Statistician/epidemiologist involved, No. (%)			
Yes	46 (46)	54 (54)	0.32
No	54 (54)	46 (46)	

Median power (interquartile range)	0.80 (0.80-0.90)	0.82 (0.80-0.90)	0.02***

Multicenter trial, No. (%)			0.001
Yes	67 (67)	86 (86)	
No	33 (33)	14 (14)	

Median sample size (interquartile range)	248 (118-633)	500 (300-900)	<0.001***

Funding source, No. (%)			0.004**
Study supported by the industry	50 (50)	70 (70)	
Study supported by institutional funds	33 (33)	25 (25)	
No funding source or not stipulated	17 (17)	5 (5)	

* Comparisons between superiority and noninferiority trials, ** Fisher's exact test, *** Mann-Whitney test

### Differences used to estimate sample size

In 161 articles (80.5%), the standardized difference in proportions or in means could be obtained directly from information provided in the methods section of the article. For 39 trials (19.5%), we imputed the standardized difference in proportions (n = 25) or in means (n = 14) using additional information (such as the sample size or observed variance estimates; see Methods).

**Figure 2:**
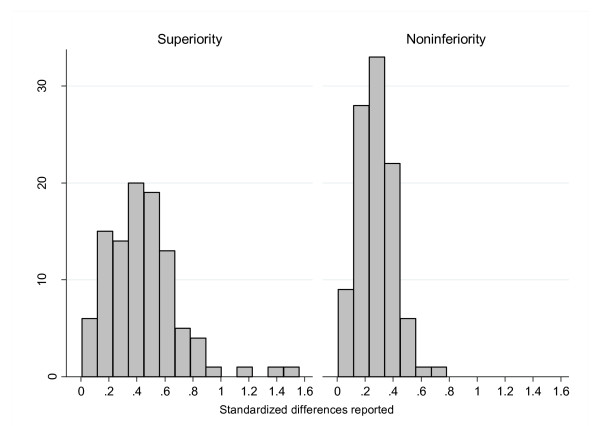
Distribution of the standardized differences in superiority and noninferiority trials.

Overall, the mean standardized difference was 0.45 for superiority trials and 0.29 for noninferiority trials (Table 2). This difference was seen for standardized differences in both means (0.56 versus 0.40) and proportions (0.37 versus 0.27). All these differences were statistically significant. The spread of standardized difference was wider in superiority trials than in noninferiority trials (Figure 2); this is also apparent from the standard deviations of the standardized differences (Table 2).

**Table 2:** Mean (±SD) standardized difference in proportions and in means in superiority and noninferiority trials.

	Superiority trials		Noninferiority trials		*P *value*
	**Mean (SD)**	**No**.	**Mean (SD)**	**No**.	

Standardized difference in proportions	0.37 (0.20)	60	0.27 (0.12)	86	0.001

Standardized difference in means	0.56 (0.30)	40	0.40 (0.11)	14	0.006

Standardized difference	0.45 (0.26)	100	0.29 (0.13)	100	<0.001

SD: standard deviation; *Student t test

### Trial characteristics associated with clinical differences used to estimate sample size

Subgroup comparisons of the standardized differences were conducted separately for superiority and noninferiority trials (Table 3). As expected, smaller detectable differences were associated with larger sample sizes and with a multicenter recruitment. Mean detectable differences were similar across years of publication, the nature of the intervention (pharmacologic or otherwise), patient age-groups, statistician involvement, and funding source, both in superiority and in noninferiority trials. However studies that used mortality as their primary outcome used smaller differences than other studies, again in both types of trials. Trials studying cardiovascular diseases used lower standardized differences than infectious diseases or other trials, particularly in nonininferiority trials. We observed the same gradient in superiority trials but due to smaller sample sizes, there was no statistical significance.

**Table 3:** Factors explaining the mean (±standard deviation) of the standardized differences in superiority and noninferiority trials and single p-value after adjustment on the type of trial.

	Superiority trials			Noninferiority trials			P-value*
		
	Standardized difference	**No**.	*P-value*	Standardized difference	**No**.	*P-value*	
Year of publication							
2004-2006	0.46 (0.23)	59	*0.42*	0.28 (0.13)	59	*0.40*	*0.72*
2007-2009	0.42 (0.30)	41	-	0.30 (0.12)	41	-	-

Medical context			*0.38*			*0.001*	*0.006*
Cardiovascular	0.36 (0.22)	14		0.18 (0.09)	19		
Infectious diseases	0.51 (0.11)	3		0.33 (0.08)	23		
Oncology	0.44 (0.23)	6		0.26 (0.008)	10		
Other	0.47 (0.27)	77		0.31 (0.14)	48		

Pharmacological trial							
Yes	0.44 (0.28)	54	*0.76*	0.29 (0.12)	73	*0.99*	*0.77*
No (other area)	0.46 (0.24)	46	-	0.29 (0.15)	27	-	-

Patients age-group			*0.43*				*0.46*
Children < 18 yrs	0.51 (0.18)	18		0.31 (0.10)	18	*0.41*	
Adults	0.44 (0.28)	77		0.28 (0.13)	82	-	
Elderly	0.35 (0.13)	5		-	0	-	

Mortality outcome							
Yes	0.28 (0.17)	11	*0.02*	0.17 (0.09)	17	*<0.001*	*<0.001*
No	0.47 (0.26)	89	-	0.31 (0.12)	83	-	-

Statistician involved							
Yes	0.44 (0.27)	46	*0.83*	0.28 (0.14)	54	*0.64*	*0.69*
No	0.45 (0.26)	54	-	0.29 (0.10)	46	-	-

Patients' recruitment							
Multicenter	0.38 (0.21)	67	*<0.001*	0.28 (0.12)	88	*0.02*	*<0.001*
Single-center	0.58 (0.30)	33	-	0.36 (0.16)	12	-	-

Funding source			*0.18*			*0.95*	*0.19*
Industry	0.40 (0.22)	50		0.29 (0.12)	70		
Institution/private	0.47 (0.32)	33		0.28 (0.14)	25		
Not reported/funded	0.53 (0.23)	17		0.29 (0.09)	5		

Sample size ≤200	0.67 (0.25) 0.42 (0.10)	37 27		0.38 (0.13) 0.36 (0.09)	10 23		
200-400	0.28 (0.07)	14	*<0.001*	0.28 (0.09)	38	*<0.001*	**
400-800	0.18 (0.13)	20		0.20 (0.14)	28		
>800							

*P-value for each factor after adjustment on the type of trial. **There was a significant interaction between superiority/noninferiority type of the trial and sample size.

Analysis of variance confirmed that the standardized difference was significantly smaller in noninferiority compared to superiority trials (adjusted mean 0.26 ± 0.03 vs. 0.39 ± 0.04, *P *< 0.001), for dichotomous compared to continuous outcomes (0.28 ± 0.02 vs. 0.42 ± 0.05, *P *< 0.001) and for mortality outcomes (adjusted mean 0.20 ± 0.05 vs. 0.39 ± 0.03; *P *= 0.01); furthermore, standardized differences tended to be higher for infectious diseases trials (0.42 ± 0.06, *P *= 0.03) and oncology trials (0.38 ± 0.05, *P *= 0.13) compared to cardiovascular diseases (0.24 ± 0.05). The adjusted R-square for this model was 0.31.

## Discussion

In this study of 200 articles published between 2004 and 2009 in 27 leading medical journals, we found that superiority trials were designed to detect larger differences than noninferiority trials. On average, trials that used dichotomous outcomes aimed to detect a mean standardized difference of 0.37 but to rule out a standardized difference of 0.27; trials that used continuous outcomes aimed to detect a standardized difference of 0.56 but to rule out a standardized difference of 0.40. Using Cohen's rule of thumb [12], superiority trials typically attempt to detect a medium effect, while inferiority trials aim to rule out a small to medium effect. This pattern is consistent with the logical requirement that for a given clinical issue, a difference between treatments that is important for clinical or public health decisions should be greater than a difference that can be deemed compatible with equality. This suggests that, in general, clinical researchers use reasonable assumptions in determining the sample size of clinical trials.

We observed a considerable variability of the differences to detect or to rule out among studies. This may be justified by the medical context. E.g., smaller differences may be relevant in noninferiority trials dealing with cardiovascular diseases than in infectious diseases or oncology, because cardiovascular disease is the main cause of mortality worldwide [13]. But patient availability may also be a factor: the potential for including patients with cardiovascular diseases into clinical trials is grater than for rare diseases, so that researchers can afford to explore smaller clinical differences. The type of outcome may also play a role; e.g., mortality is such an important outcome that trials may justifiably aim to demonstrate or exclude smaller differences than would be the case for less crucial events. However, most other factors that we examined were not associated with the differences to be detected or ruled out. Much of the observed variability remains therefore unexplained [14].

The variability in the difference to be detected or ruled out contrasts with an overwhelming consensus regarding the other parameters that guide sample size determination. Customarily, statistical tests are bilateral, type I error rates are set at 5%, and the desired power is between 80% and 90%. Thus the main reason why sample sizes vary at all is the difference to be detected (or ruled out). Whether a greater standardization of the difference to be detected is desirable is debatable. On the one hand, each research question is unique and deserves specific consideration. E.g.; a 5% improvement in mortality may not have the same relevance in an elderly population and in children. Any forceful guidelines as to the difference to be detected may promote an unreflective, cookie-cutter approach to study design. On the other hand, the absence of guidelines regarding the difference to be detected opens the door to carelessness or even to manipulation. Instead of reflecting on what would be the smallest important difference (or the largest unimportant difference), investigators may be tempted to engage themselves in the "sample size samba" [2], by retrofitting the expected detectable difference to the available number of participants. Future guidelines in this area should perhaps not provide numbers that can be plugged into sample size formulae, but rather list the salient parameters of the decision.

Several limitations of our study may be noted. Firstly, for about 20% of the trials, we recalculated the expected detectable differences but did not access the parameters actually used by authors. Secondly, we may have lacked power for subgroups analyses. As for the generalizability of our results, we included only two-arm parallel group trials with a single primary outcome. This may not be fully representative of all randomized controlled trials. As we did not perform a systematic review, but rather focused on trials published in high-profile journals, our results may not apply to all trials that are published, and even less to trials that have not been published, or have not been completed. We chose to use the standardized differences together (standardized effect sizes and standardized increments) in order to allow comparability between continuous and dichotomous outcomes [15]. Finally our study is descriptive, and does not propose a formal procedure to help researchers in the choice of differences to be detected or ruled out.

Reaching consensus on how the difference to be detected or ruled out should be chosen is an important challenge for clinical researchers. Current thinking about how results of clinical trials should be interpreted [1] (which is not the same as deciding what difference should be detected) may help guide this process. The minimal clinically important difference (MCID) is an important starting point. However, this difference typically varies from one patient to the other according to baseline risk of event, risk of complications, and individual preference [16]; in contrast, a study planner must settle on a single value. Should he or she select the mean MCID for a given population, or aim for a lower threshold, below the MCID of a large proportion (say, 80%) of the patients? Furthermore, researchers may want to detect smaller differences that would be considered meaningful for individual decision making. If a trial aims to demonstrate the potential of a new class of drugs, even a small effect may be scientifically important; a prevention trial may need to overcome the "prevention paradox", whereby a treatment brings large benefits to the community but offers little to each participating individual [17]. In other trials, researchers may aim to detect a difference that is larger than the MCID for most patients, e.g., when testing a very expensive intervention that would not be deemed cost-effective unless a large clinical benefit was demonstrated. A better consensus regarding the determination of the difference to be detected or ruled out may improve the relevance and utility of clinical trials.

## Conclusions

We see two lines for further research. One is the exploration of the researchers' reasoning that leads to the selection of a specific difference to be detected in a trial. A previous study has demonstrated a large inter-individual variability among doctors in their appreciation of the clinical relevance of treatment benefit [18], while another study has shown large differences between patients and doctors in the same area [19]. Our study shows that unexplained variability exists among published trials. We need to know what drives this variability in opinion focusing on medical area. The second line of research is the development of a consensus among researchers regarding best practices in determining the difference to be detected by a clinical trial, as stated above. We hope that our results may stimulate interest in these areas.

## Competing interests

The authors declare that they have no competing interests.

## Authors' contributions

AGA, TA, TP conceived of the study (design, search strategy of the randomized clinical trials, sampling). AGA did data collection and performed the statistical analysis. CC, DC participated to the recalculation of assumptions and to the interpretation of the data. KB participated to data collection and performed administrative, technical and material support. TP had full access to all of the data in the study and takes responsibility for their integrity of the data and the accuracy of the data analysis. AGA and TP drafted the manuscript which was commented, modified and finally approved by TA, CC, DC, KB.

All authors have read and approved the final manuscript.

## Pre-publication history

The pre-publication history for this paper can be accessed here:

http://www.biomedcentral.com/1471-2288/10/93/prepub

## Supplementary Material

Additional file 1 List of the 27 high-quality and clinical relevant journals used in the the Pubmed search. Click here for file

Additional file 2 List of the 200 randomly selected clinical trials. Click here for file
