# Supplementary material for: What differences are detected by superiority trials or ruled out by noninferiority trials? A cross-sectional study on a random sample of two-hundred two-arms parallel group randomized clinical trials
Source: BMC Med Res Methodol. 2010 Oct 15;10:93. doi: 10.1186/1471-2288-10-93 (PMC2973934; doi:10.1186/1471-2288-10-93)
Supplement: Additional file 1 — List of the 27 high-quality and clinical relevant journals used in the the Pubmed search. [file 1471-2288-10-93-S1.DOC]

**Additional file 1.** List of the 27 high-quality and clinical relevant journals used in the the Pubmed search.

| **Journal name** | **Impact factor (2008)** |
| --- | --- |
| *General and Internal medicine* |  |
| The New England journal of medicine | 50.017 |
| Lancet | 28.409 |
| JAMA | 31.718 |
| Annals of internal medicine | 17.457 |
| BMJ | 12.827 |
| Archives of internal medicine | 9.110 |
| *Oncology* |  |
| CA: A cancer journal for clinicians | 74.575 |
| Journal of clinical oncology | 17.157 |
| Lancet Oncology | 13.283 |
| *Pediatrics* |  |
| Pediatrics | 4.789 |
| Pediatric infectious disease journal | 3.176 |
| *Cardio-vascular medicine* |  |
| Circulation | 14.595 |
| American journal of cardiology | 3.905 |
| *Neurology* |  |
| Lancet Neurology | 14.270 |
| Stroke | 6.499 |
| Neurology | 7.043 |
| *Infectious diseases* |  |
| Lancet infectious diseases | 13.165 |
| Clinical infectious diseases | 8.266 |
| Journal of infectious diseases | 5.682 |
| AIDS | 5.460 |
| *Other medical specialities* |  |
| Archives of general psychiatry | 14.273 |
| Diabetes care | 7.349 |
| Critical care medicine | 6.594 |
| American journal of gastroenterology | 6.444 |
| Obstetrics and Gynecology | 4.397 |
| Chest | 5.154 |
| *Surgery* |  |
| Annals of surgery | 8.460 |
