# Supplementary material for: What differences are detected by superiority trials or ruled out by noninferiority trials? A cross-sectional study on a random sample of two-hundred two-arms parallel group randomized clinical trials
Source: BMC Med Res Methodol. 2010 Oct 15;10:93. doi: 10.1186/1471-2288-10-93 (PMC2973934; doi:10.1186/1471-2288-10-93)
Supplement: Additional file 2 — List of the 200 randomly selected clinical trials. [file 1471-2288-10-93-S2.DOC]

**Additional file 2.** List of the 200 randomly selected clinical trials.

[1-48][49-78][79-107][108-200]

1. **A randomized trial of diagnostic techniques for ventilator-associated pneumonia**. *N Engl J Med* 2006, **355**(25):2619-2630.

2. Accarino A, Perez F, Azpiroz F, Quiroga S, Malagelada JR: **Intestinal gas and bloating: effect of prokinetic stimulation**. *Am J Gastroenterol* 2008, **103**(8):2036-2042.

3. Adams HP, Jr., Effron MB, Torner J, Davalos A, Frayne J, Teal P, Leclerc J, Oemar B, Padgett L, Barnathan ES *et al*: **Emergency administration of abciximab for treatment of patients with acute ischemic stroke: results of an international phase III trial: Abciximab in Emergency Treatment of Stroke Trial (AbESTT-II)**. *Stroke* 2008, **39**(1):87-99.

4. Ahmed S, Rienstra M, Crijns HJ, Links TP, Wiesfeld AC, Hillege HL, Bosker HA, Lok DJ, Van Veldhuisen DJ, Van Gelder IC: **Continuous vs episodic prophylactic treatment with amiodarone for the prevention of atrial fibrillation: a randomized trial**. *JAMA* 2008, **300**(15):1784-1792.

5. Al RA, Unlubilgin E, Kandemir O, Yalvac S, Cakir L, Haberal A: **Intravenous versus oral iron for treatment of anemia in pregnancy: a randomized trial**. *Obstet Gynecol* 2005, **106**(6):1335-1340.

6. Albers GW, Diener HC, Frison L, Grind M, Nevinson M, Partridge S, Halperin JL, Horrow J, Olsson SB, Petersen P *et al*: **Ximelagatran vs warfarin for stroke prevention in patients with nonvalvular atrial fibrillation: a randomized trial**. *JAMA* 2005, **293**(6):690-698.

7. Anderson DR, Kahn SR, Rodger MA, Kovacs MJ, Morris T, Hirsch A, Lang E, Stiell I, Kovacs G, Dreyer J *et al*: **Computed tomographic pulmonary angiography vs ventilation-perfusion lung scanning in patients with suspected pulmonary embolism: a randomized controlled trial**. *JAMA* 2007, **298**(23):2743-2753.

8. Anderson KO, Mendoza TR, Payne R, Valero V, Palos GR, Nazario A, Richman SP, Hurley J, Gning I, Lynch GR *et al*: **Pain education for underserved minority cancer patients: a randomized controlled trial**. *J Clin Oncol* 2004, **22**(24):4918-4925.

9. Ardissino D, Cavallini C, Bramucci E, Indolfi C, Marzocchi A, Manari A, Angeloni G, Carosio G, Bonizzoni E, Colusso S *et al*: **Sirolimus-eluting vs uncoated stents for prevention of restenosis in small coronary arteries: a randomized trial**. *JAMA* 2004, **292**(22):2727-2734.

10. Bakker J, Grover R, McLuckie A, Holzapfel L, Andersson J, Lodato R, Watson D, Grossman S, Donaldson J, Takala J: **Administration of the nitric oxide synthase inhibitor NG-methyl-L-arginine hydrochloride (546C88) by intravenous infusion for up to 72 hours can promote the resolution of shock in patients with severe sepsis: results of a randomized, double-blind, placebo-controlled multicenter study (study no. 144-002)**. *Crit Care Med* 2004, **32**(1):1-12.

11. Ballantyne CM, Weiss R, Moccetti T, Vogt A, Eber B, Sosef F, Duffield E: **Efficacy and safety of rosuvastatin 40 mg alone or in combination with ezetimibe in patients at high risk of cardiovascular disease (results from the EXPLORER study)**. *Am J Cardiol* 2007, **99**(5):673-680.

12. Bansch D, Steffgen F, Gronefeld G, Wolpert C, Bocker D, Mletzko RU, Schols W, Seidl K, Piel M, Ouyang F *et al*: **The 1+1 trial: a prospective trial of a dual- versus a single-chamber implantable defibrillator in patients with slow ventricular tachycardias**. *Circulation* 2004, **110**(9):1022-1029.

13. Barber MD, Kleeman S, Karram MM, Paraiso MF, Walters MD, Vasavada S, Ellerkmann M: **Transobturator tape compared with tension-free vaginal tape for the treatment of stress urinary incontinence: a randomized controlled trial**. *Obstet Gynecol* 2008, **111**(3):611-621.

14. Barnard ND, Cohen J, Jenkins DJ, Turner-McGrievy G, Gloede L, Jaster B, Seidl K, Green AA, Talpers S: **A low-fat vegan diet improves glycemic control and cardiovascular risk factors in a randomized clinical trial in individuals with type 2 diabetes**. *Diabetes Care* 2006, **29**(8):1777-1783.

15. Barnett AH, Dreyer M, Lange P, Serdarevic-Pehar M: **An open, randomized, parallel-group study to compare the efficacy and safety profile of inhaled human insulin (Exubera) with metformin as adjunctive therapy in patients with type 2 diabetes poorly controlled on a sulfonylurea**. *Diabetes Care* 2006, **29**(6):1282-1287.

16. Barnhart KT, Rosenberg MJ, MacKay HT, Blithe DL, Higgins J, Walsh T, Wan L, Thomas M, Creinin MD, Westhoff C *et al*: **Contraceptive efficacy of a novel spermicidal microbicide used with a diaphragm: a randomized controlled trial**. *Obstet Gynecol* 2007, **110**(3):577-586.

17. Barter PJ, Caulfield M, Eriksson M, Grundy SM, Kastelein JJ, Komajda M, Lopez-Sendon J, Mosca L, Tardif JC, Waters DD *et al*: **Effects of torcetrapib in patients at high risk for coronary events**. *N Engl J Med* 2007, **357**(21):2109-2122.

18. Bellomo R, Morimatsu H, French C, Cole L, Story D, Uchino S, Naka T: **The effects of saline or albumin resuscitation on acid-base status and serum electrolytes**. *Crit Care Med* 2006, **34**(12):2891-2897.

19. Benson CA, van der Horst C, Lamarca A, Haas DW, McDonald CK, Steinhart CR, Rublein J, Quinn JB, Mondou E, Rousseau F: **A randomized study of emtricitabine and lamivudine in stably suppressed patients with HIV**. *AIDS* 2004, **18**(17):2269-2276.

20. Berenguer J, Gonzalez J, Ribera E, Domingo P, Santos J, Miralles P, Angels Ribas M, Asensi V, Gimeno JL, Perez-Molina JA *et al*: **Didanosine, lamivudine, and efavirenz versus zidovudine, lamivudine, and efavirenz for the initial treatment of HIV type 1 infection: final analysis (48 weeks) of a prospective, randomized, noninferiority clinical trial, GESIDA 3903**. *Clin Infect Dis* 2008, **47**(8):1083-1092.

21. Bernardi E, Camporese G, Buller HR, Siragusa S, Imberti D, Berchio A, Ghirarduzzi A, Verlato F, Anastasio R, Prati C *et al*: **Serial 2-point ultrasonography plus D-dimer vs whole-leg color-coded Doppler ultrasonography for diagnosing suspected symptomatic deep vein thrombosis: a randomized controlled trial**. *JAMA* 2008, **300**(14):1653-1659.

22. Bertrand OF, De Larochelliere R, Rodes-Cabau J, Proulx G, Gleeton O, Nguyen CM, Dery JP, Barbeau G, Noel B, Larose E *et al*: **A randomized study comparing same-day home discharge and abciximab bolus only to overnight hospitalization and abciximab bolus and infusion after transradial coronary stent implantation**. *Circulation* 2006, **114**(24):2636-2643.

23. Black SB, Cimino CO, Hansen J, Lewis E, Ray P, Corsaro B, Graepel J, Laufer D: **Immunogenicity and safety of measles-mumps-rubella, varicella and Haemophilus influenzae type b vaccines administered concurrently with a fourth dose of heptavalent pneumococcal conjugate vaccine compared with the vaccines administered without heptavalent pneumococcal conjugate vaccine**. *Pediatr Infect Dis J* 2006, **25**(4):306-311.

24. Blazing MA, de Lemos JA, White HD, Fox KA, Verheugt FW, Ardissino D, DiBattiste PM, Palmisano J, Bilheimer DW, Snapinn SM *et al*: **Safety and efficacy of enoxaparin vs unfractionated heparin in patients with non-ST-segment elevation acute coronary syndromes who receive tirofiban and aspirin: a randomized controlled trial**. *JAMA* 2004, **292**(1):55-64.

25. Bollschweiler E, Apitzsch J, Obliers R, Koerfer A, Monig SP, Metzger R, Holscher AH: **Improving informed consent of surgical patients using a multimedia-based program? Results of a prospective randomized multicenter study of patients before cholecystectomy**. *Ann Surg* 2008, **248**(2):205-211.

26. Bonello L, Camoin-Jau L, Armero S, Com O, Arques S, Burignat-Bonello C, Giacomoni MP, Bonello R, Collet F, Rossi P *et al*: **Tailored clopidogrel loading dose according to platelet reactivity monitoring to prevent acute and subacute stent thrombosis**. *Am J Cardiol* 2009, **103**(1):5-10.

27. Boutis K, Willan AR, Babyn P, Narayanan UG, Alman B, Schuh S: **A randomized, controlled trial of a removable brace versus casting in children with low-risk ankle fractures**. *Pediatrics* 2007, **119**(6):e1256-1263.

28. Bow EJ, Rotstein C, Noskin GA, Laverdiere M, Schwarer AP, Segal BH, Seymour JF, Szer J, Sanche S: **A randomized, open-label, multicenter comparative study of the efficacy and safety of piperacillin-tazobactam and cefepime for the empirical treatment of febrile neutropenic episodes in patients with hematologic malignancies**. *Clin Infect Dis* 2006, **43**(4):447-459.

29. Bradley JS, Arguedas A, Blumer JL, Saez-Llorens X, Melkote R, Noel GJ: **Comparative study of levofloxacin in the treatment of children with community-acquired pneumonia**. *Pediatr Infect Dis J* 2007, **26**(10):868-878.

30. Brodie MJ, Perucca E, Ryvlin P, Ben-Menachem E, Meencke HJ: **Comparison of levetiracetam and controlled-release carbamazepine in newly diagnosed epilepsy**. *Neurology* 2007, **68**(6):402-408.

31. Brouwer CN, Maille AR, Rovers MM, Veenhoven RH, Grobbee DE, Sanders EA, Schilder AG: **Effect of pneumococcal vaccination on quality of life in children with recurrent acute otitis media: a randomized, controlled trial**. *Pediatrics* 2005, **115**(2):273-279.

32. Brunkhorst FM, Engel C, Bloos F, Meier-Hellmann A, Ragaller M, Weiler N, Moerer O, Gruendling M, Oppert M, Grond S *et al*: **Intensive insulin therapy and pentastarch resuscitation in severe sepsis**. *N Engl J Med* 2008, **358**(2):125-139.

33. Buller HR, Cohen AT, Davidson B, Decousus H, Gallus AS, Gent M, Pillion G, Piovella F, Prins MH, Raskob GE: **Idraparinux versus standard therapy for venous thromboembolic disease**. *N Engl J Med* 2007, **357**(11):1094-1104.

34. Burgess IF, Brown CM, Lee PN: **Treatment of head louse infestation with 4% dimeticone lotion: randomised controlled equivalence trial**. *BMJ* 2005, **330**(7505):1423.

35. Buyon JP, Petri MA, Kim MY, Kalunian KC, Grossman J, Hahn BH, Merrill JT, Sammaritano L, Lockshin M, Alarcon GS *et al*: **The effect of combined estrogen and progesterone hormone replacement therapy on disease activity in systemic lupus erythematosus: a randomized trial**. *Ann Intern Med* 2005, **142**(12 Pt 1):953-962.

36. Campbell IA, Bentley DP, Prescott RJ, Routledge PA, Shetty HG, Williamson IJ: **Anticoagulation for three versus six months in patients with deep vein thrombosis or pulmonary embolism, or both: randomised trial**. *BMJ* 2007, **334**(7595):674.

37. Cannon CP, Braunwald E, McCabe CH, Rader DJ, Rouleau JL, Belder R, Joyal SV, Hill KA, Pfeffer MA, Skene AM: **Intensive versus moderate lipid lowering with statins after acute coronary syndromes**. *N Engl J Med* 2004, **350**(15):1495-1504.

38. Carbajal R, Lenclen R, Jugie M, Paupe A, Barton BA, Anand KJ: **Morphine does not provide adequate analgesia for acute procedural pain among preterm neonates**. *Pediatrics* 2005, **115**(6):1494-1500.

39. Cardinale D, Colombo A, Sandri MT, Lamantia G, Colombo N, Civelli M, Martinelli G, Veglia F, Fiorentini C, Cipolla CM: **Prevention of high-dose chemotherapy-induced cardiotoxicity in high-risk patients by angiotensin-converting enzyme inhibition**. *Circulation* 2006, **114**(23):2474-2481.

40. Chang TT, Gish RG, de Man R, Gadano A, Sollano J, Chao YC, Lok AS, Han KH, Goodman Z, Zhu J *et al*: **A comparison of entecavir and lamivudine for HBeAg-positive chronic hepatitis B**. *N Engl J Med* 2006, **354**(10):1001-1010.

41. Ciarlet M, Sani-Grosso R, Yuan G, Liu GF, Heaton PM, Gottesdiener KM, Arredondo JL, Schodel F: **Concomitant use of the oral pentavalent human-bovine reassortant rotavirus vaccine and oral poliovirus vaccine**. *Pediatr Infect Dis J* 2008, **27**(10):874-880.

42. Clegg HW, Ryan AG, Dallas SD, Kaplan EL, Johnson DR, Norton HJ, Roddey OF, Martin ES, Swetenburg RL, Koonce EW *et al*: **Treatment of streptococcal pharyngitis with once-daily compared with twice-daily amoxicillin: a noninferiority trial**. *Pediatr Infect Dis J* 2006, **25**(9):761-767.

43. Cluett ER, Pickering RM, Getliffe K, St George Saunders NJ: **Randomised controlled trial of labouring in water compared with standard of augmentation for management of dystocia in first stage of labour**. *BMJ* 2004, **328**(7435):314.

44. Conrad SA, Gabrielli A, Margolis B, Quartin A, Hata JS, Frank WO, Bagin RG, Rock JA, Hepburn B, Laine L: **Randomized, double-blind comparison of immediate-release omeprazole oral suspension versus intravenous cimetidine for the prevention of upper gastrointestinal bleeding in critically ill patients**. *Crit Care Med* 2005, **33**(4):760-765.

45. Conter V, Valsecchi MG, Silvestri D, Campbell M, Dibar E, Magyarosy E, Gadner H, Stary J, Benoit Y, Zimmermann M *et al*: **Pulses of vincristine and dexamethasone in addition to intensive chemotherapy for children with intermediate-risk acute lymphoblastic leukaemia: a multicentre randomised trial**. *Lancet* 2007, **369**(9556):123-131.

46. Cortot A, Maetz D, Degoutte E, Delette O, Meunier P, Tan G, Cazals JB, Dewit O, Hebuterne X, Beorchia S *et al*: **Mesalamine foam enema versus mesalamine liquid enema in active left-sided ulcerative colitis**. *Am J Gastroenterol* 2008, **103**(12):3106-3114.

47. Crawford BA, Kam C, Pavlovic J, Byth K, Handelsman DJ, Angus PW, McCaughan GW: **Zoledronic acid prevents bone loss after liver transplantation: a randomized, double-blind, placebo-controlled trial**. *Ann Intern Med* 2006, **144**(4):239-248.

48. Creinin MD, Fox MC, Teal S, Chen A, Schaff EA, Meyn LA: **A randomized comparison of misoprostol 6 to 8 hours versus 24 hours after mifepristone for abortion**. *Obstet Gynecol* 2004, **103**(5 Pt 1):851-859.

49. Creinin MD, Schlaff W, Archer DF, Wan L, Frezieres R, Thomas M, Rosenberg M, Higgins J: **Progesterone receptor modulator for emergency contraception: a randomized controlled trial**. *Obstet Gynecol* 2006, **108**(5):1089-1097.

50. Creinin MD, Schreiber CA, Bednarek P, Lintu H, Wagner MS, Meyn LA: **Mifepristone and misoprostol administered simultaneously versus 24 hours apart for abortion: a randomized controlled trial**. *Obstet Gynecol* 2007, **109**(4):885-894.

51. Cryer DR, Nicholas SP, Henry DH, Mills DJ, Stadel BV: **Comparative outcomes study of metformin intervention versus conventional approach the COSMIC Approach Study**. *Diabetes Care* 2005, **28**(3):539-543.

52. Dabydeen L, Thomas JE, Aston TJ, Hartley H, Sinha SK, Eyre JA: **High-energy and -protein diet increases brain and corticospinal tract growth in term and preterm infants after perinatal brain injury**. *Pediatrics* 2008, **121**(1):148-156.

53. Daley AJ, Crank H, Saxton JM, Mutrie N, Coleman R, Roalfe A: **Randomized trial of exercise therapy in women treated for breast cancer**. *J Clin Oncol* 2007, **25**(13):1713-1721.

54. Darboe MK, Thurnham DI, Morgan G, Adegbola RA, Secka O, Solon JA, Jackson SJ, Northrop-Clewes C, Fulford TJ, Doherty CP *et al*: **Effectiveness of an early supplementation scheme of high-dose vitamin A versus standard WHO protocol in Gambian mothers and infants: a randomised controlled trial**. *Lancet* 2007, **369**(9579):2088-2096.

55. de Kraker J, Graf N, van Tinteren H, Pein F, Sandstedt B, Godzinski J, Tournade MF: **Reduction of postoperative chemotherapy in children with stage I intermediate-risk and anaplastic Wilms' tumour (SIOP 93-01 trial): a randomised controlled trial**. *Lancet* 2004, **364**(9441):1229-1235.

56. DeJesus E, Herrera G, Teofilo E, Gerstoft J, Buendia CB, Brand JD, Brothers CH, Hernandez J, Castillo SA, Bonny T *et al*: **Abacavir versus zidovudine combined with lamivudine and efavirenz, for the treatment of antiretroviral-naive HIV-infected adults**. *Clin Infect Dis* 2004, **39**(7):1038-1046.

57. Dempsey AF, Zimet GD, Davis RL, Koutsky L: **Factors that are associated with parental acceptance of human papillomavirus vaccines: a randomized intervention study of written information about HPV**. *Pediatrics* 2006, **117**(5):1486-1493.

58. Dennehy PH, Bertrand HR, Silas PE, Damaso S, Friedland LR, Abu-Elyazeed R: **Coadministration of RIX4414 oral human rotavirus vaccine does not impact the immune response to antigens contained in routine infant vaccines in the United States**. *Pediatrics* 2008, **122**(5):e1062-1066.

59. Di Bisceglie AM, Shiffman ML, Everson GT, Lindsay KL, Everhart JE, Wright EC, Lee WM, Lok AS, Bonkovsky HL, Morgan TR *et al*: **Prolonged therapy of advanced chronic hepatitis C with low-dose peginterferon**. *N Engl J Med* 2008, **359**(23):2429-2441.

60. Dibra A, Kastrati A, Mehilli J, Pache J, Schuhlen H, von Beckerath N, Ulm K, Wessely R, Dirschinger J, Schomig A: **Paclitaxel-eluting or sirolimus-eluting stents to prevent restenosis in diabetic patients**. *N Engl J Med* 2005, **353**(7):663-670.

61. Diener HC, Ringelstein EB, von Kummer R, Landgraf H, Koppenhagen K, Harenberg J, Rektor I, Csanyi A, Schneider D, Klingelhofer J *et al*: **Prophylaxis of thrombotic and embolic events in acute ischemic stroke with the low-molecular-weight heparin certoparin: results of the PROTECT Trial**. *Stroke* 2006, **37**(1):139-144.

62. Dodd JM, Crowther CA, Robinson JS: **Morning compared with evening induction of labor: a nested randomized controlled trial. A nested randomized controlled trial**. *Obstet Gynecol* 2006, **108**(2):350-360.

63. Eckardt JR, von Pawel J, Papai Z, Tomova A, Tzekova V, Crofts TE, Brannon S, Wissel P, Ross G: **Open-label, multicenter, randomized, phase III study comparing oral topotecan/cisplatin versus etoposide/cisplatin as treatment for chemotherapy-naive patients with extensive-disease small-cell lung cancer**. *J Clin Oncol* 2006, **24**(13):2044-2051.

64. Ell C, Fischbach W, Bronisch HJ, Dertinger S, Layer P, Runzi M, Schneider T, Kachel G, Gruger J, Kollinger M *et al*: **Randomized trial of low-volume PEG solution versus standard PEG + electrolytes for bowel cleansing before colonoscopy**. *Am J Gastroenterol* 2008, **103**(4):883-893.

65. Ellis DA, Frey MA, Naar-King S, Templin T, Cunningham P, Cakan N: **Use of multisystemic therapy to improve regimen adherence among adolescents with type 1 diabetes in chronic poor metabolic control: a randomized controlled trial**. *Diabetes Care* 2005, **28**(7):1604-1610.

66. Engler RJ, Nelson MR, Klote MM, VanRaden MJ, Huang CY, Cox NJ, Klimov A, Keitel WA, Nichol KL, Carr WW *et al*: **Half- vs full-dose trivalent inactivated influenza vaccine (2004-2005): age, dose, and sex effects on immune responses**. *Arch Intern Med* 2008, **168**(22):2405-2414.

67. Feagan BG, Sandborn WJ, Mittmann U, Bar-Meir S, D'Haens G, Bradette M, Cohen A, Dallaire C, Ponich TP, McDonald JW *et al*: **Omega-3 free fatty acids for the maintenance of remission in Crohn disease: the EPIC Randomized Controlled Trials**. *JAMA* 2008, **299**(14):1690-1697.

68. Feldman EJ, Brandwein J, Stone R, Kalaycio M, Moore J, O'Connor J, Wedel N, Roboz GJ, Miller C, Chopra R *et al*: **Phase III randomized multicenter study of a humanized anti-CD33 monoclonal antibody, lintuzumab, in combination with chemotherapy, versus chemotherapy alone in patients with refractory or first-relapsed acute myeloid leukemia**. *J Clin Oncol* 2005, **23**(18):4110-4116.

69. Fiessinger JN, Huisman MV, Davidson BL, Bounameaux H, Francis CW, Eriksson H, Lundstrom T, Berkowitz SD, Nystrom P, Thorsen M *et al*: **Ximelagatran vs low-molecular-weight heparin and warfarin for the treatment of deep vein thrombosis: a randomized trial**. *JAMA* 2005, **293**(6):681-689.

70. Figueras J, Llado L, Ruiz D, Ramos E, Busquets J, Rafecas A, Torras J, Fabregat J: **Complete versus selective portal triad clamping for minor liver resections: a prospective randomized trial**. *Ann Surg* 2005, **241**(4):582-590.

71. Fossati R, Apolone G, Negri E, Compagnoni A, La Vecchia C, Mangano S, Clivio L, Garattini S: **A double-blind, placebo-controlled, randomized trial of bupropion for smoking cessation in primary care**. *Arch Intern Med* 2007, **167**(16):1791-1797.

72. Fowler VG, Jr., Boucher HW, Corey GR, Abrutyn E, Karchmer AW, Rupp ME, Levine DP, Chambers HF, Tally FP, Vigliani GA *et al*: **Daptomycin versus standard therapy for bacteremia and endocarditis caused by Staphylococcus aureus**. *N Engl J Med* 2006, **355**(7):653-665.

73. Gallant JE, DeJesus E, Arribas JR, Pozniak AL, Gazzard B, Campo RE, Lu B, McColl D, Chuck S, Enejosa J *et al*: **Tenofovir DF, emtricitabine, and efavirenz vs. zidovudine, lamivudine, and efavirenz for HIV**. *N Engl J Med* 2006, **354**(3):251-260.

74. Garcia Garcia ML, Wahn U, Gilles L, Swern A, Tozzi CA, Polos P: **Montelukast, compared with fluticasone, for control of asthma among 6- to 14-year-old patients with mild asthma: the MOSAIC study**. *Pediatrics* 2005, **116**(2):360-369.

75. Garg A, Butz AM, Dworkin PH, Lewis RA, Thompson RE, Serwint JR: **Improving the management of family psychosocial problems at low-income children's well-child care visits: the WE CARE Project**. *Pediatrics* 2007, **120**(3):547-558.

76. Geyer JR, Sposto R, Jennings M, Boyett JM, Axtell RA, Breiger D, Broxson E, Donahue B, Finlay JL, Goldwein JW *et al*: **Multiagent chemotherapy and deferred radiotherapy in infants with malignant brain tumors: a report from the Children's Cancer Group**. *J Clin Oncol* 2005, **23**(30):7621-7631.

77. Gielen AC, McKenzie LB, McDonald EM, Shields WC, Wang MC, Cheng YJ, Weaver NL, Walker AR: **Using a computer kiosk to promote child safety: results of a randomized, controlled trial in an urban pediatric emergency department**. *Pediatrics* 2007, **120**(2):330-339.

78. Giesen-Bloo J, van Dyck R, Spinhoven P, van Tilburg W, Dirksen C, van Asselt T, Kremers I, Nadort M, Arntz A: **Outpatient psychotherapy for borderline personality disorder: randomized trial of schema-focused therapy vs transference-focused psychotherapy**. *Arch Gen Psychiatry* 2006, **63**(6):649-658.

79. Ginzler EM, Dooley MA, Aranow C, Kim MY, Buyon J, Merrill JT, Petri M, Gilkeson GS, Wallace DJ, Weisman MH *et al*: **Mycophenolate mofetil or intravenous cyclophosphamide for lupus nephritis**. *N Engl J Med* 2005, **353**(21):2219-2228.

80. Glaspy J, Vadhan-Raj S, Patel R, Bosserman L, Hu E, Lloyd RE, Boccia RV, Tomita D, Rossi G: **Randomized comparison of every-2-week darbepoetin alfa and weekly epoetin alfa for the treatment of chemotherapy-induced anemia: the 20030125 Study Group Trial**. *J Clin Oncol* 2006, **24**(15):2290-2297.

81. Glauser TA, Ayala R, Elterman RD, Mitchell WG, Van Orman CB, Gauer LJ, Lu Z: **Double-blind placebo-controlled trial of adjunctive levetiracetam in pediatric partial seizures**. *Neurology* 2006, **66**(11):1654-1660.

82. Goldberg RB, Fonseca VA, Truitt KE, Jones MR: **Efficacy and safety of colesevelam in patients with type 2 diabetes mellitus and inadequate glycemic control receiving insulin-based therapy**. *Arch Intern Med* 2008, **168**(14):1531-1540.

83. Green MC, Buzdar AU, Smith T, Ibrahim NK, Valero V, Rosales MF, Cristofanilli M, Booser DJ, Pusztai L, Rivera E *et al*: **Weekly paclitaxel improves pathologic complete remission in operable breast cancer when compared with paclitaxel once every 3 weeks**. *J Clin Oncol* 2005, **23**(25):5983-5992.

84. Greenspan SL, Bone HG, Ettinger MP, Hanley DA, Lindsay R, Zanchetta JR, Blosch CM, Mathisen AL, Morris SA, Marriott TB: **Effect of recombinant human parathyroid hormone (1-84) on vertebral fracture and bone mineral density in postmenopausal women with osteoporosis: a randomized trial**. *Ann Intern Med* 2007, **146**(5):326-339.

85. Greenspan SL, Nelson JB, Trump DL, Resnick NM: **Effect of once-weekly oral alendronate on bone loss in men receiving androgen deprivation therapy for prostate cancer: a randomized trial**. *Ann Intern Med* 2007, **146**(6):416-424.

86. Gueugniaud PY, David JS, Chanzy E, Hubert H, Dubien PY, Mauriaucourt P, Braganca C, Billeres X, Clotteau-Lambert MP, Fuster P *et al*: **Vasopressin and epinephrine vs. epinephrine alone in cardiopulmonary resuscitation**. *N Engl J Med* 2008, **359**(1):21-30.

87. Hadigan C, Yawetz S, Thomas A, Havers F, Sax PE, Grinspoon S: **Metabolic effects of rosiglitazone in HIV lipodystrophy: a randomized, controlled trial**. *Ann Intern Med* 2004, **140**(10):786-794.

88. Hay EM, Mullis R, Lewis M, Vohora K, Main CJ, Watson P, Dziedzic KS, Sim J, Minns Lowe C, Croft PR: **Comparison of physical treatments versus a brief pain-management programme for back pain in primary care: a randomised clinical trial in physiotherapy practice**. *Lancet* 2005, **365**(9476):2024-2030.

89. Heal C, Buettner P, Raasch B, Browning S, Graham D, Bidgood R, Campbell M, Cruikshank R: **Can sutures get wet? Prospective randomised controlled trial of wound management in general practice**. *BMJ* 2006, **332**(7549):1053-1056.

90. Heine RJ, Van Gaal LF, Johns D, Mihm MJ, Widel MH, Brodows RG: **Exenatide versus insulin glargine in patients with suboptimally controlled type 2 diabetes: a randomized trial**. *Ann Intern Med* 2005, **143**(8):559-569.

91. Heyde GS, Koch KT, de Winter RJ, Dijkgraaf MG, Klees MI, Dijksman LM, Piek JJ, Tijssen JG: **Randomized trial comparing same-day discharge with overnight hospital stay after percutaneous coronary intervention: results of the Elective PCI in Outpatient Study (EPOS)**. *Circulation* 2007, **115**(17):2299-2306.

92. Hohnloser SH, Kuck KH, Dorian P, Roberts RS, Hampton JR, Hatala R, Fain E, Gent M, Connolly SJ: **Prophylactic use of an implantable cardioverter-defibrillator after acute myocardial infarction**. *N Engl J Med* 2004, **351**(24):2481-2488.

93. Holmes C, Wilkinson D, Dean C, Vethanayagam S, Olivieri S, Langley A, Pandita-Gunawardena ND, Hogg F, Clare C, Damms J: **The efficacy of donepezil in the treatment of neuropsychiatric symptoms in Alzheimer disease**. *Neurology* 2004, **63**(2):214-219.

94. Home PD, Pocock SJ, Beck-Nielsen H, Gomis R, Hanefeld M, Jones NP, Komajda M, McMurray JJ: **Rosiglitazone evaluated for cardiovascular outcomes--an interim analysis**. *N Engl J Med* 2007, **357**(1):28-38.

95. Jaksic B, Martinelli G, Perez-Oteyza J, Hartman CS, Leonard LB, Tack KJ: **Efficacy and safety of linezolid compared with vancomycin in a randomized, double-blind study of febrile neutropenic patients with cancer**. *Clin Infect Dis* 2006, **42**(5):597-607.

96. Jauregui LE, Babazadeh S, Seltzer E, Goldberg L, Krievins D, Frederick M, Krause D, Satilovs I, Endzinas Z, Breaux J *et al*: **Randomized, double-blind comparison of once-weekly dalbavancin versus twice-daily linezolid therapy for the treatment of complicated skin and skin structure infections**. *Clin Infect Dis* 2005, **41**(10):1407-1415.

97. Jenkins DJ, Kendall CW, McKeown-Eyssen G, Josse RG, Silverberg J, Booth GL, Vidgen E, Josse AR, Nguyen TH, Corrigan S *et al*: **Effect of a low-glycemic index or a high-cereal fiber diet on type 2 diabetes: a randomized trial**. *JAMA* 2008, **300**(23):2742-2753.

98. Johnston SL, Blasi F, Black PN, Martin RJ, Farrell DJ, Nieman RB: **The effect of telithromycin in acute exacerbations of asthma**. *N Engl J Med* 2006, **354**(15):1589-1600.

99. Kane RL, Wang J, Garrard J: **Reporting in randomized clinical trials improved after adoption of the CONSORT statement**. *J Clin Epidemiol* 2007, **60**(3):241-249.

100. Kearon C, Ginsberg JS, Julian JA, Douketis J, Solymoss S, Ockelford P, Jackson S, Turpie AG, MacKinnon B, Hirsh J *et al*: **Comparison of fixed-dose weight-adjusted unfractionated heparin and low-molecular-weight heparin for acute treatment of venous thromboembolism**. *JAMA* 2006, **296**(8):935-942.

101. Kim YW, Baik YH, Yun YH, Nam BH, Kim DH, Choi IJ, Bae JM: **Improved quality of life outcomes after laparoscopy-assisted distal gastrectomy for early gastric cancer: results of a prospective randomized clinical trial**. *Ann Surg* 2008, **248**(5):721-727.

102. Kulnigg S, Stoinov S, Simanenkov V, Dudar LV, Karnafel W, Garcia LC, Sambuelli AM, D'Haens G, Gasche C: **A novel intravenous iron formulation for treatment of anemia in inflammatory bowel disease: the ferric carboxymaltose (FERINJECT) randomized controlled trial**. *Am J Gastroenterol* 2008, **103**(5):1182-1192.

103. Lacroix J, Hebert PC, Hutchison JS, Hume HA, Tucci M, Ducruet T, Gauvin F, Collet JP, Toledano BJ, Robillard P *et al*: **Transfusion strategies for patients in pediatric intensive care units**. *N Engl J Med* 2007, **356**(16):1609-1619.

104. Lai CL, Gane E, Liaw YF, Hsu CW, Thongsawat S, Wang Y, Chen Y, Heathcote EJ, Rasenack J, Bzowej N *et al*: **Telbivudine versus lamivudine in patients with chronic hepatitis B**. *N Engl J Med* 2007, **357**(25):2576-2588.

105. Langley JM, Halperin SA, Boucher FD, Smith B: **Azithromycin is as effective as and better tolerated than erythromycin estolate for the treatment of pertussis**. *Pediatrics* 2004, **114**(1):e96-101.

106. Lautrette A, Darmon M, Megarbane B, Joly LM, Chevret S, Adrie C, Barnoud D, Bleichner G, Bruel C, Choukroun G *et al*: **A communication strategy and brochure for relatives of patients dying in the ICU**. *N Engl J Med* 2007, **356**(5):469-478.

107. Leroy O, Saux P, Bedos JP, Caulin E: **Comparison of levofloxacin and cefotaxime combined with ofloxacin for ICU patients with community-acquired pneumonia who do not require vasopressors**. *Chest* 2005, **128**(1):172-183.

108. Lim SG, Ng TM, Kung N, Krastev Z, Volfova M, Husa P, Lee SS, Chan S, Shiffman ML, Washington MK *et al*: **A double-blind placebo-controlled study of emtricitabine in chronic hepatitis B**. *Arch Intern Med* 2006, **166**(1):49-56.

109. Linden HM, Haskell CM, Green SJ, Osborne CK, Sledge GW, Jr., Shapiro CL, Ingle JN, Lew D, Hutchins LF, Livingston RB *et al*: **Sequenced compared with simultaneous anthracycline and cyclophosphamide in high-risk stage I and II breast cancer: final analysis from INT-0137 (S9313)**. *J Clin Oncol* 2007, **25**(6):656-661.

110. Lipsky BA, Holroyd KJ, Zasloff M: **Topical versus systemic antimicrobial therapy for treating mildly infected diabetic foot ulcers: a randomized, controlled, double-blinded, multicenter trial of pexiganan cream**. *Clin Infect Dis* 2008, **47**(12):1537-1545.

111. Lukka H, Hayter C, Julian JA, Warde P, Morris WJ, Gospodarowicz M, Levine M, Sathya J, Choo R, Prichard H *et al*: **Randomized trial comparing two fractionation schedules for patients with localized prostate cancer**. *J Clin Oncol* 2005, **23**(25):6132-6138.

112. Maltais F, Bourbeau J, Shapiro S, Lacasse Y, Perrault H, Baltzan M, Hernandez P, Rouleau M, Julien M, Parenteau S *et al*: **Effects of home-based pulmonary rehabilitation in patients with chronic obstructive pulmonary disease: a randomized trial**. *Ann Intern Med* 2008, **149**(12):869-878.

113. Manzoni P, Mostert M, Leonessa ML, Priolo C, Farina D, Monetti C, Latino MA, Gomirato G: **Oral supplementation with Lactobacillus casei subspecies rhamnosus prevents enteric colonization by Candida species in preterm neonates: a randomized study**. *Clin Infect Dis* 2006, **42**(12):1735-1742.

114. Marson A, Jacoby A, Johnson A, Kim L, Gamble C, Chadwick D: **Immediate versus deferred antiepileptic drug treatment for early epilepsy and single seizures: a randomised controlled trial**. *Lancet* 2005, **365**(9476):2007-2013.

115. Mas JL, Chatellier G, Beyssen B, Branchereau A, Moulin T, Becquemin JP, Larrue V, Lievre M, Leys D, Bonneville JF *et al*: **Endarterectomy versus stenting in patients with symptomatic severe carotid stenosis**. *N Engl J Med* 2006, **355**(16):1660-1671.

116. McPherson AC, Glazebrook C, Forster D, James C, Smyth A: **A randomized, controlled trial of an interactive educational computer package for children with asthma**. *Pediatrics* 2006, **117**(4):1046-1054.

117. Mehilli J, Kastrati A, Wessely R, Dibra A, Hausleiter J, Jaschke B, Dirschinger J, Schomig A: **Randomized trial of a nonpolymer-based rapamycin-eluting stent versus a polymer-based paclitaxel-eluting stent for the reduction of late lumen loss**. *Circulation* 2006, **113**(2):273-279.

118. Mercuri E, Bertini E, Messina S, Solari A, D'Amico A, Angelozzi C, Battini R, Berardinelli A, Boffi P, Bruno C *et al*: **Randomized, double-blind, placebo-controlled trial of phenylbutyrate in spinal muscular atrophy**. *Neurology* 2007, **68**(1):51-55.

119. Merten GJ, Burgess WP, Gray LV, Holleman JH, Roush TS, Kowalchuk GJ, Bersin RM, Van Moore A, Simonton CA, 3rd, Rittase RA *et al*: **Prevention of contrast-induced nephropathy with sodium bicarbonate: a randomized controlled trial**. *JAMA* 2004, **291**(19):2328-2334.

120. Murray CS, Woodcock A, Langley SJ, Morris J, Custovic A: **Secondary prevention of asthma by the use of Inhaled Fluticasone propionate in Wheezy INfants (IFWIN): double-blind, randomised, controlled study**. *Lancet* 2006, **368**(9537):754-762.

121. Nickel MK, Krawczyk J, Nickel C, Forthuber P, Kettler C, Leiberich P, Muehlbacher M, Tritt K, Mitterlehner FO, Lahmann C *et al*: **Anger, interpersonal relationships, and health-related quality of life in bullying boys who are treated with outpatient family therapy: a randomized, prospective, controlled trial with 1 year of follow-up**. *Pediatrics* 2005, **116**(2):e247-254.

122. Noel GJ, Blumer JL, Pichichero ME, Hedrick JA, Schwartz RH, Balis DA, Melkote R, Bagchi P, Arguedas A: **A randomized comparative study of levofloxacin versus amoxicillin/clavulanate for treatment of infants and young children with recurrent or persistent acute otitis media**. *Pediatr Infect Dis J* 2008, **27**(6):483-489.

123. Olshansky B, Day JD, Moore S, Gering L, Rosenbaum M, McGuire M, Brown S, Lerew DR: **Is dual-chamber programming inferior to single-chamber programming in an implantable cardioverter-defibrillator? Results of the INTRINSIC RV (Inhibition of Unnecessary RV Pacing With AVSH in ICDs) study**. *Circulation* 2007, **115**(1):9-16.

124. Paavonen J, Jenkins D, Bosch FX, Naud P, Salmeron J, Wheeler CM, Chow SN, Apter DL, Kitchener HC, Castellsague X *et al*: **Efficacy of a prophylactic adjuvanted bivalent L1 virus-like-particle vaccine against infection with human papillomavirus types 16 and 18 in young women: an interim analysis of a phase III double-blind, randomised controlled trial**. *Lancet* 2007, **369**(9580):2161-2170.

125. Pantoni L, del Ser T, Soglian AG, Amigoni S, Spadari G, Binelli D, Inzitari D: **Efficacy and safety of nimodipine in subcortical vascular dementia: a randomized placebo-controlled trial**. *Stroke* 2005, **36**(3):619-624.

126. Paradise JL, Feldman HM, Campbell TF, Dollaghan CA, Rockette HE, Pitcairn DL, Smith CG, Colborn DK, Bernard BS, Kurs-Lasky M *et al*: **Tympanostomy tubes and developmental outcomes at 9 to 11 years of age**. *N Engl J Med* 2007, **356**(3):248-261.

127. Paridaens RJ, Dirix LY, Beex LV, Nooij M, Cameron DA, Cufer T, Piccart MJ, Bogaerts J, Therasse P: **Phase III study comparing exemestane with tamoxifen as first-line hormonal treatment of metastatic breast cancer in postmenopausal women: the European Organisation for Research and Treatment of Cancer Breast Cancer Cooperative Group**. *J Clin Oncol* 2008, **26**(30):4883-4890.

128. Park JO, Kim SW, Ahn JS, Suh C, Lee JS, Jang JS, Cho EK, Yang SH, Choi JH, Heo DS *et al*: **Phase III trial of two versus four additional cycles in patients who are nonprogressive after two cycles of platinum-based chemotherapy in non small-cell lung cancer**. *J Clin Oncol* 2007, **25**(33):5233-5239.

129. Peeters KC, Marijnen CA, Nagtegaal ID, Kranenbarg EK, Putter H, Wiggers T, Rutten H, Pahlman L, Glimelius B, Leer JW *et al*: **The TME trial after a median follow-up of 6 years: increased local control but no survival benefit in irradiated patients with resectable rectal carcinoma**. *Ann Surg* 2007, **246**(5):693-701.

130. Petersen L, Jeppesen P, Thorup A, Abel MB, Ohlenschlaeger J, Christensen TO, Krarup G, Jorgensen P, Nordentoft M: **A randomised multicentre trial of integrated versus standard treatment for patients with a first episode of psychotic illness**. *BMJ* 2005, **331**(7517):602.

131. Petri M, Kim MY, Kalunian KC, Grossman J, Hahn BH, Sammaritano LR, Lockshin M, Merrill JT, Belmont HM, Askanase AD *et al*: **Combined oral contraceptives in women with systemic lupus erythematosus**. *N Engl J Med* 2005, **353**(24):2550-2558.

132. Peul WC, van Houwelingen HC, van den Hout WB, Brand R, Eekhof JA, Tans JT, Thomeer RT, Koes BW: **Surgery versus prolonged conservative treatment for sciatica**. *N Engl J Med* 2007, **356**(22):2245-2256.

133. Porschen R, Arkenau HT, Kubicka S, Greil R, Seufferlein T, Freier W, Kretzschmar A, Graeven U, Grothey A, Hinke A *et al*: **Phase III study of capecitabine plus oxaliplatin compared with fluorouracil and leucovorin plus oxaliplatin in metastatic colorectal cancer: a final report of the AIO Colorectal Study Group**. *J Clin Oncol* 2007, **25**(27):4217-4223.

134. Prince RL, Austin N, Devine A, Dick IM, Bruce D, Zhu K: **Effects of ergocalciferol added to calcium on the risk of falls in elderly high-risk women**. *Arch Intern Med* 2008, **168**(1):103-108.

135. Puhan MA, Busching G, Schunemann HJ, VanOort E, Zaugg C, Frey M: **Interval versus continuous high-intensity exercise in chronic obstructive pulmonary disease: a randomized trial**. *Ann Intern Med* 2006, **145**(11):816-825.

136. Pulido F, Arribas JR, Delgado R, Cabrero E, Gonzalez-Garcia J, Perez-Elias MJ, Arranz A, Portilla J, Pasquau J, Iribarren JA *et al*: **Lopinavir-ritonavir monotherapy versus lopinavir-ritonavir and two nucleosides for maintenance therapy of HIV**. *AIDS* 2008, **22**(2):F1-9.

137. Ramage JI, Jr., Rumalla A, Baron TH, Pochron NL, Zinsmeister AR, Murray JA, Norton ID, Diehl N, Romero Y: **A prospective, randomized, double-blind, placebo-controlled trial of endoscopic steroid injection therapy for recalcitrant esophageal peptic strictures**. *Am J Gastroenterol* 2005, **100**(11):2419-2425.

138. Ramharter M, Oyakhirome S, Klein Klouwenberg P, Adegnika AA, Agnandji ST, Missinou MA, Matsiegui PB, Mordmuller B, Borrmann S, Kun JF *et al*: **Artesunate-clindamycin versus quinine-clindamycin in the treatment of Plasmodium falciparum malaria: a randomized controlled trial**. *Clin Infect Dis* 2005, **40**(12):1777-1784.

139. Ramlau R, Gervais R, Krzakowski M, von Pawel J, Kaukel E, Abratt RP, Dharan B, Grotzinger KM, Ross G, Dane G *et al*: **Phase III study comparing oral topotecan to intravenous docetaxel in patients with pretreated advanced non-small-cell lung cancer**. *J Clin Oncol* 2006, **24**(18):2800-2807.

140. Reboli AC, Rotstein C, Pappas PG, Chapman SW, Kett DH, Kumar D, Betts R, Wible M, Goldstein BP, Schranz J *et al*: **Anidulafungin versus fluconazole for invasive candidiasis**. *N Engl J Med* 2007, **356**(24):2472-2482.

141. Riedner G, Rusizoka M, Todd J, Maboko L, Hoelscher M, Mmbando D, Samky E, Lyamuya E, Mabey D, Grosskurth H *et al*: **Single-dose azithromycin versus penicillin G benzathine for the treatment of early syphilis**. *N Engl J Med* 2005, **353**(12):1236-1244.

142. Roed HG, Langkilde A, Sellebjerg F, Lauritzen M, Bang P, Morup A, Frederiksen JL: **A double-blind, randomized trial of IV immunoglobulin treatment in acute optic neuritis**. *Neurology* 2005, **64**(5):804-810.

143. Rosenstock J, Ahmann AJ, Colon G, Scism-Bacon J, Jiang H, Martin S: **Advancing insulin therapy in type 2 diabetes previously treated with glargine plus oral agents: prandial premixed (insulin lispro protamine suspension/lispro) versus basal/bolus (glargine/lispro) therapy**. *Diabetes Care* 2008, **31**(1):20-25.

144. Rosenstock J, Baron MA, Dejager S, Mills D, Schweizer A: **Comparison of vildagliptin and rosiglitazone monotherapy in patients with type 2 diabetes: a 24-week, double-blind, randomized trial**. *Diabetes Care* 2007, **30**(2):217-223.

145. Roy-Byrne PP, Craske MG, Stein MB, Sullivan G, Bystritsky A, Katon W, Golinelli D, Sherbourne CD: **A randomized effectiveness trial of cognitive-behavioral therapy and medication for primary care panic disorder**. *Arch Gen Psychiatry* 2005, **62**(3):290-298.

146. Rozendaal RM, Koes BW, van Osch GJ, Uitterlinden EJ, Garling EH, Willemsen SP, Ginai AZ, Verhaar JA, Weinans H, Bierma-Zeinstra SM: **Effect of glucosamine sulfate on hip osteoarthritis: a randomized trial**. *Ann Intern Med* 2008, **148**(4):268-277.

147. Sagiv R, Sadan O, Boaz M, Dishi M, Schechter E, Golan A: **A new approach to office hysteroscopy compared with traditional hysteroscopy: a randomized controlled trial**. *Obstet Gynecol* 2006, **108**(2):387-392.

148. Saha D, Karim MM, Khan WA, Ahmed S, Salam MA, Bennish ML: **Single-dose azithromycin for the treatment of cholera in adults**. *N Engl J Med* 2006, **354**(23):2452-2462.

149. Salvarani C, Macchioni P, Manzini C, Paolazzi G, Trotta A, Manganelli P, Cimmino M, Gerli R, Catanoso MG, Boiardi L *et al*: **Infliximab plus prednisone or placebo plus prednisone for the initial treatment of polymyalgia rheumatica: a randomized trial**. *Ann Intern Med* 2007, **146**(9):631-639.

150. Schaefer-Graf UM, Kjos SL, Fauzan OH, Buhling KJ, Siebert G, Buhrer C, Ladendorf B, Dudenhausen JW, Vetter K: **A randomized trial evaluating a predominantly fetal growth-based strategy to guide management of gestational diabetes in Caucasian women**. *Diabetes Care* 2004, **27**(2):297-302.

151. Schanler RJ, Lau C, Hurst NM, Smith EO: **Randomized trial of donor human milk versus preterm formula as substitutes for mothers' own milk in the feeding of extremely premature infants**. *Pediatrics* 2005, **116**(2):400-406.

152. Schnipper JL, Kirwin JL, Cotugno MC, Wahlstrom SA, Brown BA, Tarvin E, Kachalia A, Horng M, Roy CL, McKean SC *et al*: **Role of pharmacist counseling in preventing adverse drug events after hospitalization**. *Arch Intern Med* 2006, **166**(5):565-571.

153. Shaw LJ, Berman DS, Maron DJ, Mancini GB, Hayes SW, Hartigan PM, Weintraub WS, O'Rourke RA, Dada M, Spertus JA *et al*: **Optimal medical therapy with or without percutaneous coronary intervention to reduce ischemic burden: results from the Clinical Outcomes Utilizing Revascularization and Aggressive Drug Evaluation (COURAGE) trial nuclear substudy**. *Circulation* 2008, **117**(10):1283-1291.

154. Sherman DG, Albers GW, Bladin C, Fieschi C, Gabbai AA, Kase CS, O'Riordan W, Pineo GF: **The efficacy and safety of enoxaparin versus unfractionated heparin for the prevention of venous thromboembolism after acute ischaemic stroke (PREVAIL Study): an open-label randomised comparison**. *Lancet* 2007, **369**(9570):1347-1355.

155. Shiffman ML, Suter F, Bacon BR, Nelson D, Harley H, Sola R, Shafran SD, Barange K, Lin A, Soman A *et al*: **Peginterferon alfa-2a and ribavirin for 16 or 24 weeks in HCV genotype 2 or 3**. *N Engl J Med* 2007, **357**(2):124-134.

156. Shuaib A, Lees KR, Lyden P, Grotta J, Davalos A, Davis SM, Diener HC, Ashwood T, Wasiewski WW, Emeribe U: **NXY-059 for the treatment of acute ischemic stroke**. *N Engl J Med* 2007, **357**(6):562-571.

157. Silberstein SD, Mannix LK, Goldstein J, Couch JR, Byrd SC, Ames MH, McDonald SA, Lener SE, Toso C: **Multimechanistic (sumatriptan-naproxen) early intervention for the acute treatment of migraine**. *Neurology* 2008, **71**(2):114-121.

158. Singh AK, Szczech L, Tang KL, Barnhart H, Sapp S, Wolfson M, Reddan D: **Correction of anemia with epoetin alfa in chronic kidney disease**. *N Engl J Med* 2006, **355**(20):2085-2098.

159. Sinha SK, Lacaze-Masmonteil T, Valls i Soler A, Wiswell TE, Gadzinowski J, Hajdu J, Bernstein G, Sanchez-Luna M, Segal R, Schaber CJ *et al*: **A multicenter, randomized, controlled trial of lucinactant versus poractant alfa among very premature infants at high risk for respiratory distress syndrome**. *Pediatrics* 2005, **115**(4):1030-1038.

160. Skyler JS, Krischer JP, Wolfsdorf J, Cowie C, Palmer JP, Greenbaum C, Cuthbertson D, Rafkin-Mervis LE, Chase HP, Leschek E: **Effects of oral insulin in relatives of patients with type 1 diabetes: The Diabetes Prevention Trial--Type 1**. *Diabetes Care* 2005, **28**(5):1068-1076.

161. Smith C, Crowther C, Willson K, Hotham N, McMillian V: **A randomized controlled trial of ginger to treat nausea and vomiting in pregnancy**. *Obstet Gynecol* 2004, **103**(4):639-645.

162. Smyth A, Tan KH, Hyman-Taylor P, Mulheran M, Lewis S, Stableforth D, Prof Knox A: **Once versus three-times daily regimens of tobramycin treatment for pulmonary exacerbations of cystic fibrosis--the TOPIC study: a randomised controlled trial**. *Lancet* 2005, **365**(9459):573-578.

163. Spandorfer PR, Alessandrini EA, Joffe MD, Localio R, Shaw KN: **Oral versus intravenous rehydration of moderately dehydrated children: a randomized, controlled trial**. *Pediatrics* 2005, **115**(2):295-301.

164. Stahl M, Stuschke M, Lehmann N, Meyer HJ, Walz MK, Seeber S, Klump B, Budach W, Teichmann R, Schmitt M *et al*: **Chemoradiation with and without surgery in patients with locally advanced squamous cell carcinoma of the esophagus**. *J Clin Oncol* 2005, **23**(10):2310-2317.

165. Stankoff B, Waubant E, Confavreux C, Edan G, Debouverie M, Rumbach L, Moreau T, Pelletier J, Lubetzki C, Clanet M: **Modafinil for fatigue in MS: a randomized placebo-controlled double-blind study**. *Neurology* 2005, **64**(7):1139-1143.

166. Stellbrink C, Nixdorff U, Hofmann T, Lehmacher W, Daniel WG, Hanrath P, Geller C, Mugge A, Sehnert W, Schmidt-Lucke C *et al*: **Safety and efficacy of enoxaparin compared with unfractionated heparin and oral anticoagulants for prevention of thromboembolic complications in cardioversion of nonvalvular atrial fibrillation: the Anticoagulation in Cardioversion using Enoxaparin (ACE) trial**. *Circulation* 2004, **109**(8):997-1003.

167. Stockler MR, O'Connell R, Nowak AK, Goldstein D, Turner J, Wilcken NR, Wyld D, Abdi EA, Glasgow A, Beale PJ *et al*: **Effect of sertraline on symptoms and survival in patients with advanced cancer, but without major depression: a placebo-controlled double-blind randomised trial**. *Lancet Oncol* 2007, **8**(7):603-612.

168. Stone GW, Bertrand ME, Moses JW, Ohman EM, Lincoff AM, Ware JH, Pocock SJ, McLaurin BT, Cox DA, Jafar MZ *et al*: **Routine upstream initiation vs deferred selective use of glycoprotein IIb/IIIa inhibitors in acute coronary syndromes: the ACUITY Timing trial**. *JAMA* 2007, **297**(6):591-602.

169. Stone GW, Midei M, Newman W, Sanz M, Hermiller JB, Williams J, Farhat N, Mahaffey KW, Cutlip DE, Fitzgerald PJ *et al*: **Comparison of an everolimus-eluting stent and a paclitaxel-eluting stent in patients with coronary artery disease: a randomized trial**. *JAMA* 2008, **299**(16):1903-1913.

170. Strong V, Waters R, Hibberd C, Murray G, Wall L, Walker J, McHugh G, Walker A, Sharpe M: **Management of depression for people with cancer (SMaRT oncology 1): a randomised trial**. *Lancet* 2008, **372**(9632):40-48.

171. Studer UE, Whelan P, Albrecht W, Casselman J, de Reijke T, Hauri D, Loidl W, Isorna S, Sundaram SK, Debois M *et al*: **Immediate or deferred androgen deprivation for patients with prostate cancer not suitable for local treatment with curative intent: European Organisation for Research and Treatment of Cancer (EORTC) Trial 30891**. *J Clin Oncol* 2006, **24**(12):1868-1876.

172. Sundar S, Jha TK, Thakur CP, Sinha PK, Bhattacharya SK: **Injectable paromomycin for Visceral leishmaniasis in India**. *N Engl J Med* 2007, **356**(25):2571-2581.

173. Suzuki T, Matsushima M, Masui A, Watanabe K, Takagi A, Ogawa Y, Shirai T, Mine T: **Effect of Helicobacter pylori eradication in patients with chronic idiopathic thrombocytopenic purpura-a randomized controlled trial**. *Am J Gastroenterol* 2005, **100**(6):1265-1270.

174. te Pas AB, Walther FJ: **A randomized, controlled trial of delivery-room respiratory management in very preterm infants**. *Pediatrics* 2007, **120**(2):322-329.

175. Tejedor JC, Omenaca F, Garcia-Sicilia J, Verdaguer J, Van Esso D, Esporrin C, Molina V, Muro M, Mares J, Enrubia M *et al*: **Immunogenicity and reactogenicity of a three-dose primary vaccination course with a combined diphtheria-tetanus-acellular pertussis-hepatitis B-inactivated polio-haemophilus influenzae type b vaccine coadministered with a meningococcal C conjugate vaccine**. *Pediatr Infect Dis J* 2004, **23**(12):1109-1115.

176. ten Dam VH, Box FM, de Craen AJ, van den Heuvel DM, Bollen EL, Murray HM, van Buchem MA, Westendorp RG, Blauw GJ: **Lack of effect of pravastatin on cerebral blood flow or parenchymal volume loss in elderly at risk for vascular disease**. *Stroke* 2005, **36**(8):1633-1636.

177. Thurin A, Hausken J, Hillensjo T, Jablonowska B, Pinborg A, Strandell A, Bergh C: **Elective single-embryo transfer versus double-embryo transfer in in vitro fertilization**. *N Engl J Med* 2004, **351**(23):2392-2402.

178. Todd MM, Hindman BJ, Clarke WR, Torner JC: **Mild intraoperative hypothermia during surgery for intracranial aneurysm**. *N Engl J Med* 2005, **352**(2):135-145.

179. Torres A, Garau J, Arvis P, Carlet J, Choudhri S, Kureishi A, Le Berre MA, Lode H, Winter J, Read RC: **Moxifloxacin monotherapy is effective in hospitalized patients with community-acquired pneumonia: the MOTIV study--a randomized clinical trial**. *Clin Infect Dis* 2008, **46**(10):1499-1509.

180. Twelves C, Wong A, Nowacki MP, Abt M, Burris H, 3rd, Carrato A, Cassidy J, Cervantes A, Fagerberg J, Georgoulias V *et al*: **Capecitabine as adjuvant treatment for stage III colon cancer**. *N Engl J Med* 2005, **352**(26):2696-2704.

181. Valgimigli M, Campo G, Percoco G, Bolognese L, Vassanelli C, Colangelo S, de Cesare N, Rodriguez AE, Ferrario M, Moreno R *et al*: **Comparison of angioplasty with infusion of tirofiban or abciximab and with implantation of sirolimus-eluting or uncoated stents for acute myocardial infarction: the MULTISTRATEGY randomized trial**. *JAMA* 2008, **299**(15):1788-1799.

182. van Hilten JA, van de Watering LM, van Bockel JH, van de Velde CJ, Kievit J, Brand R, van den Hout WB, Geelkerken RH, Roumen RM, Wesselink RM *et al*: **Effects of transfusion with red cells filtered to remove leucocytes: randomised controlled trial in patients undergoing major surgery**. *BMJ* 2004, **328**(7451):1281.

183. van Mastrigt GA, Heijmans J, Severens JL, Fransen EJ, Roekaerts P, Voss G, Maessen JG: **Short-stay intensive care after coronary artery bypass surgery: randomized clinical trial on safety and cost-effectiveness**. *Crit Care Med* 2006, **34**(1):65-75.

184. Victor JC, Monto AS, Surdina TY, Suleimenova SZ, Vaughan G, Nainan OV, Favorov MO, Margolis HS, Bell BP: **Hepatitis A vaccine versus immune globulin for postexposure prophylaxis**. *N Engl J Med* 2007, **357**(17):1685-1694.

185. Villareal DT, Banks M, Sinacore DR, Siener C, Klein S: **Effect of weight loss and exercise on frailty in obese older adults**. *Arch Intern Med* 2006, **166**(8):860-866.

186. Viscusi ER, Reynolds L, Chung F, Atkinson LE, Khanna S: **Patient-controlled transdermal fentanyl hydrochloride vs intravenous morphine pump for postoperative pain: a randomized controlled trial**. *JAMA* 2004, **291**(11):1333-1341.

187. Walders N, Kercsmar C, Schluchter M, Redline S, Kirchner HL, Drotar D: **An interdisciplinary intervention for undertreated pediatric asthma**. *Chest* 2006, **129**(2):292-299.

188. Walsh TJ, Teppler H, Donowitz GR, Maertens JA, Baden LR, Dmoszynska A, Cornely OA, Bourque MR, Lupinacci RJ, Sable CA *et al*: **Caspofungin versus liposomal amphotericin B for empirical antifungal therapy in patients with persistent fever and neutropenia**. *N Engl J Med* 2004, **351**(14):1391-1402.

189. Walter EB, Neuzil KM, Zhu Y, Fairchok MP, Gagliano ME, Monto AS, Englund JA: **Influenza vaccine immunogenicity in 6- to 23-month-old children: are identical antigens necessary for priming?** *Pediatrics* 2006, **118**(3):e570-578.

190. Warriner IK, Meirik O, Hoffman M, Morroni C, Harries J, My Huong NT, Vy ND, Seuc AH: **Rates of complication in first-trimester manual vacuum aspiration abortion done by doctors and mid-level providers in South Africa and Vietnam: a randomised controlled equivalence trial**. *Lancet* 2006, **368**(9551):1965-1972.

191. Welte T, Petermann W, Schurmann D, Bauer TT, Reimnitz P: **Treatment with sequential intravenous or oral moxifloxacin was associated with faster clinical improvement than was standard therapy for hospitalized patients with community-acquired pneumonia who received initial parenteral therapy**. *Clin Infect Dis* 2005, **41**(12):1697-1705.

192. Werdan K, Pilz G, Muller-Werdan U, Maas Enriquez M, Schmitt DV, Mohr FW, Neeser G, Schondube F, Schafers HJ, Haverich A *et al*: **Immunoglobulin G treatment of postcardiac surgery patients with score-identified severe systemic inflammatory response syndrome--the ESSICS study**. *Crit Care Med* 2008, **36**(3):716-723.

193. Wilcox MH, Tack KJ, Bouza E, Herr DL, Ruf BR, Ijzerman MM, Croos-Dabrera RV, Kunkel MJ, Knirsch C: **Complicated skin and skin-structure infections and catheter-related bloodstream infections: noninferiority of linezolid in a phase 3 study**. *Clin Infect Dis* 2009, **48**(2):203-212.

194. Willenheimer R, van Veldhuisen DJ, Silke B, Erdmann E, Follath F, Krum H, Ponikowski P, Skene A, van de Ven L, Verkenne P *et al*: **Effect on survival and hospitalization of initiating treatment for chronic heart failure with bisoprolol followed by enalapril, as compared with the opposite sequence: results of the randomized Cardiac Insufficiency Bisoprolol Study (CIBIS) III**. *Circulation* 2005, **112**(16):2426-2435.

195. Winblad B, Kilander L, Eriksson S, Minthon L, Batsman S, Wetterholm AL, Jansson-Blixt C, Haglund A: **Donepezil in patients with severe Alzheimer's disease: double-blind, parallel-group, placebo-controlled study**. *Lancet* 2006, **367**(9516):1057-1065.

196. Wong CA, Scavone BM, Peaceman AM, McCarthy RJ, Sullivan JT, Diaz NT, Yaghmour E, Marcus RJ, Sherwani SS, Sproviero MT *et al*: **The risk of cesarean delivery with neuraxial analgesia given early versus late in labor**. *N Engl J Med* 2005, **352**(7):655-665.

197. Yonkers KA, Brown C, Pearlstein TB, Foegh M, Sampson-Landers C, Rapkin A: **Efficacy of a new low-dose oral contraceptive with drospirenone in premenstrual dysphoric disorder**. *Obstet Gynecol* 2005, **106**(3):492-501.

198. Zaninotto G, Annese V, Costantini M, Del Genio A, Costantino M, Epifani M, Gatto G, D'Onofrio V, Benini L, Contini S *et al*: **Randomized controlled trial of botulinum toxin versus laparoscopic heller myotomy for esophageal achalasia**. *Ann Surg* 2004, **239**(3):364-370.

199. Zeiher BG, Artigas A, Vincent JL, Dmitrienko A, Jackson K, Thompson BT, Bernard G: **Neutrophil elastase inhibition in acute lung injury: results of the STRIVE study**. *Crit Care Med* 2004, **32**(8):1695-1702.

200. Zongo I, Dorsey G, Rouamba N, Tinto H, Dokomajilar C, Guiguemde RT, Rosenthal PJ, Ouedraogo JB: **Artemether-lumefantrine versus amodiaquine plus sulfadoxine-pyrimethamine for uncomplicated falciparum malaria in Burkina Faso: a randomised non-inferiority trial**. *Lancet* 2007, **369**(9560):491-498.
